# Microbial dysbiosis and the aging process: a review on the potential age-deceleration role of *Lactiplantibacillus plantarum*

**DOI:** 10.3389/fmicb.2024.1260793

**Published:** 2024-02-19

**Authors:** Nishant Gupta, N. S. Abd El-Gawaad, L. O. Mallasiy, Harsh Gupta, Virendra Kumar Yadav, Saad Alghamdi, Naeem F. Qusty

**Affiliations:** ^1^Medical Research and Development, River Engineering, Noida, India; ^2^Department of Physics, Faculty of Science, King Khalid University, Abha, Saudi Arabia; ^3^Department of Home Economics, Faculty of Science and Arts in Tihama, King Khalid University, Muhayil, Saudi Arabia; ^4^ITCS, Karnal, Haryana, India; ^5^Hemchandracharya North Gujarat University, Patan, Gujarat, India; ^6^Department of Clinical Laboratory Sciences, Faculty of Applied Medical Sciences, University of Umm Al-Qura University, Makkah, Saudi Arabia

**Keywords:** microbial dysbiosis, probiotic, photo-aging, anti-oxidative stress pathway, *Lactiplantibacillus plantarums* strains, collagen improvement

## Abstract

Gut microbiota dysbiosis has been a serious risk factor for several gastric and systemic diseases. Recently, gut microbiota’s role in aging was discussed. Available preclinical evidence suggests that the probiotic bacteria *Lactiplantibacillus plantarum*s (LP) may influence the aging process via modulation of the gut microbiota. The present review summarized compelling evidence of LP’s potential effect on aging hallmarks such as oxidative stress, inflammation, DNA methylation, and mitochondrial dysfunction. LP gavage modulates gut microbiota and improves overall endurance in aging animal models. LP cell constituents exert considerable antioxidant potential which may reduce ROS levels directly. In addition, restored gut microbiota facilitate a healthy intestinal milieu and accelerate multi-channel communication via signaling factors such as SCFA and GABA. Signaling factors further activate specific transcription factor Nrf2 in order to reduce oxidative damage. Nrf2 regulates cellular defense systems involving anti-inflammatory cytokines, MMPs, and protective enzymes against MAPKs. We concluded that LP supplementation may be an effective approach to managing aging and associated health risks.

## Graphical abstract

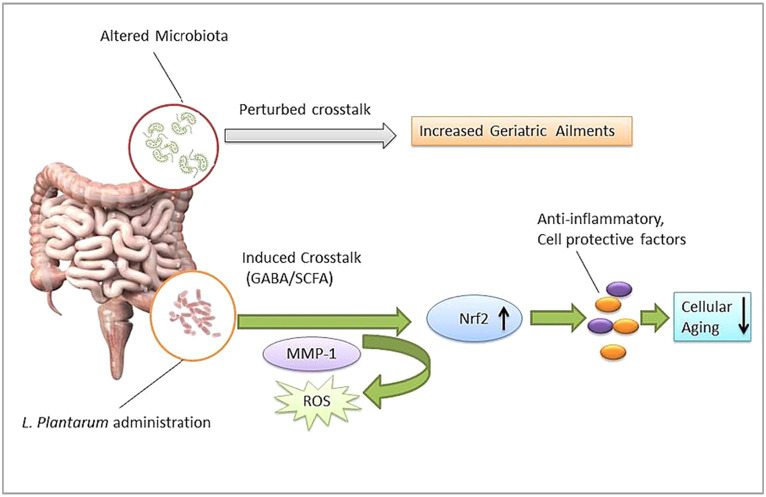

## 1 Introduction

The human gut microbiota (GM) plays a crucial role in multiple physiological activities, so a healthy composition of GM has been a widely discussed subject for overall health. Factors such as diet, environment, and inappropriate medication (antibiotics and xenobiotics) may alter the intrinsic composition of gut microbiota and induce microbial dysbiosis-associated health anomalies including arthritis, atherosclerosis, cirrhosis, intestinal cancer, hypertension, and diabetes (Li et al., 2017; Gupta et al., 2022). Growing evidence indicates gut microbiome dysbiosis may influence aging and associated health complications. Hitherto, little is understood about the significance of human GM in the complex aging process (Maynard and Weinkove, 2018; Ragonnaud and Biragyn, 2021; Ghosh et al., 2022). Aging is an inevitable, multifactorial complex process that influences almost every organ and tissue (Campisi et al., 2019; Schumacher et al., 2021). GM’s role in aging may be a helpful approach to influencing and understanding the other dimensions of the aging process. Microbial therapeutic interventions such as probiotics have been the most promising agents in restoring those guts-friendly bacteria, in cases of repeated infectious diarrheas and irritable bowel syndrome; in recent years, the use of probiotics has been widened to many other health conditions such as lowering debilitating side effects of radio and chemotherapy. The concept of ‘bugs to drugs’ seems relevant as the use of probiotics extends (O’Hara and Shanahan, 2007; Vivarelli et al., 2019; Rahman et al., 2022).

The identification or development of new probiotics has been an alluring arena of microbiology. Members of *Lactobacillaceae* have been important probiotic agents (Azad et al., 2018). Probiotics associated with several actions of mechanism, such as the host’s innate and adaptive immune modulation, considerable anti-inflammatory effects by TLR4/TLR2/MyD88/NFkB signaling, decreased gut permeability, and alterations in inflammasome, have been proposed by researchers (Van Zyl et al., 2020). *Lactobacilli* species are known to produce proteins p40 and p75 in the host, which showed immunomodulation; they trans-activated the epidermal growth factor receptor (EGFR) in intestinal epithelial cells of the host, and antimicrobial factors such as Aggregation-promoting factor (APF) and Bacteriocins decreased pathogens such as *Salmonella typhimurium*, *Clostridium sporogenes,* and *Enterococcus faecalis* colonization in host (García-Peña et al., 2017; Du et al., 2021). In particular, *Lactobacillus plantarum* (LP) or *Lactiplantibacillus plantarum* (updated name) has been a well-studied probiotic model subjected to multiple health effects such as anti-microbial, anti-cancerous, and anti-inflammatory. LP supplementation associated with positive outcomes is observed in bowel disease, atopic dermatitis, intestinal infections, and maternal and neonatal hematological issues. LP is one of the most plausible lactic acid bacterium species found in diverse ecological conditions (Seddik et al., 2017; Zhou et al., 2021; Jeong et al., 2023; OjiNjideka Hemphill et al., 2023). LP’s effect on aging is in the initial stage (Lin et al., 2021; Ding et al., 2022; Kumar et al., 2022). Available studies showed LP may influence longevity and healthy aging via enhancing gut and host integrity. However, the role of LP supplementation in aging and associated health conditions is scarcely understood so far (Biagi et al., 2012). This review discusses the significance of LP supplementation in managing signs of aging-associated health complications.

## 2 Materials and method (searching strategies)

**Figure 1:**
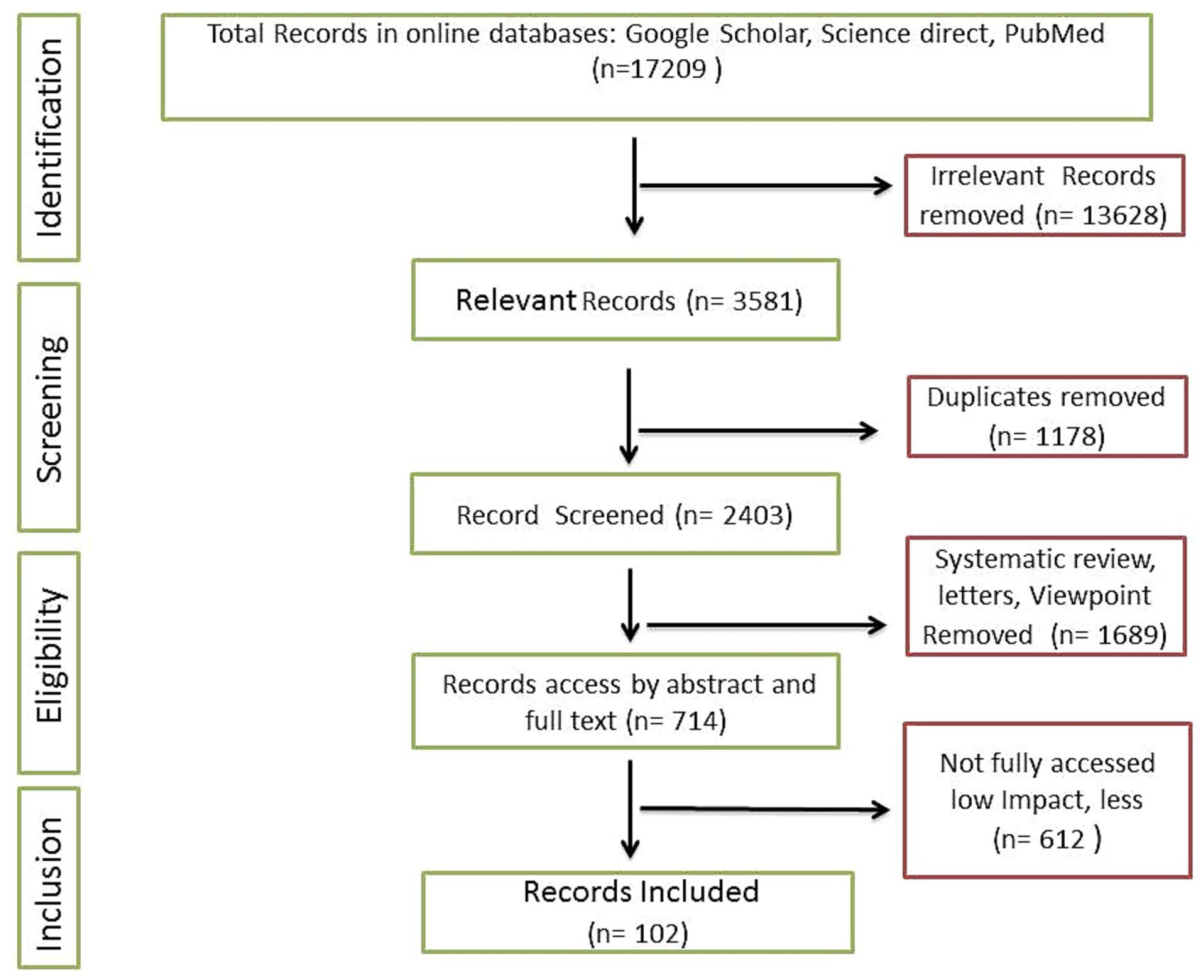
Summary of the methodology.

The present review is comprised of recent relevant articles including pre-clinical and clinical studies. Searching strategies mainly include online research databases such as Google Scholar, PubMed, and Science Direct. Overall, 17,209 results were obtained when specific filters such as time period (2015 to 2023) and keywords (‘Probiotic *Lactobacillus plantarum*’, ‘*Lactobacillus plantarum* and aging’, ‘*Lactobacillus plantarum* effect on aging’, ‘*Lactobacillus plantarum* medicinal uses’) were applied. More specific keywords were also recruited to fill the potential gap. Figures and illustrative works were original and designed according to compiled research outcomes, and in case of retrieval, significant modifications were employed. For instance, some vector images were retrieved from Pixabay under the no attribution required category and modified completely (99%) using tools such as Adobe Photoshop and Microsoft PowerPoint. Articles selection and shortlisting criteria were based on the significant impact factors and citations of relevant publications (Figure 1). A total of 645 relevant articles were selected initially. Duplicate and irrelevant articles were removed. Finally, 112 articles were incorporated in the present review.

## 3 Gut microbiota, microbial dysbiosis, and aging

**Figure 2:**
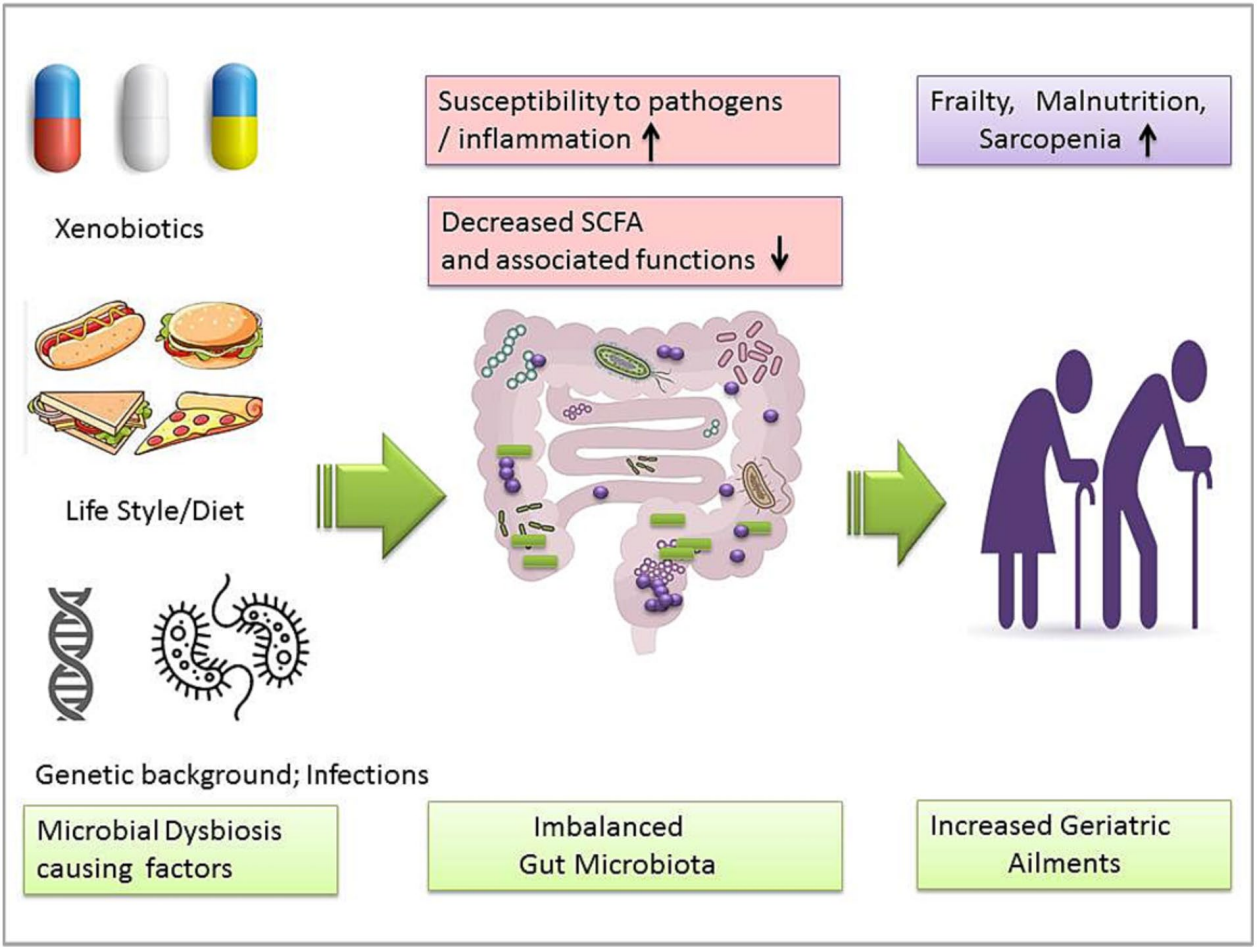
Gut microbiota dysbiosis-causing factors and imbalanced microbiota-associated geriatric ailments.

Humans have been reviewed as ‘metaorganisms’ due to their integral symbiotic association with the intestinal microbiota (Dam et al., 2019). Noticeably, most diseases might be linked to gut microbiota dysbiosis (Langille et al., 2014). Evidence of aging and microbiome association is also growing (Lustgarten, 2016; Wei et al., 2022). Perturbed GM composition could be responsible for several geriatric degenerative diseases such as Parkinson’s disease, Alzheimer’s disease, sarcopenia, and physical frailty (Figure 2; Lakshminarayanan et al., 2014; Nadeem et al., 2015; Mossad et al., 2021). GM’s role in the aging process could be important in minimizing the risk of aging-associated ailments. However, the diversity and specificity of the human gut microbiome have been influenced by multiple intrinsic and extrinsic factors. A cohort analysis showed that gut microbiome biodiversity is positively associated with age in young adults in the United Kingdom, the United States, and Colombia (Heintz and Mair, 2014). It was observed that 16S rRNA gene sequencing of the infant to elderly macaques microbiome indicates microbiome composition variation and connectivity responsible for age-dependent network changes and altered metabolic functions of amino acids, carbohydrates, lipids, and phenotypes in the microbial community (De la Cuesta-Zuluaga et al., 2019). Moreover, abnormal DNA methylation, characterized by gradual genome demethylation and hypermethylation was observed during the aging process. Usually, DNA methylation is an inevitable normal process that occurs throughout the lifetime. However, why changes occur in DNA methylation is not explained in detail. DNA methylation is susceptible to external factors such as environment, smoking, and lifestyle. So, DNA methylation biomarkers may predict tissue’s biological age across the human lifespan, including development (Jung and Pfeifer, 2015; Zampieri et al., 2015; Salameh et al., 2020). GM’s role in DNA methylation is insufficiently understood. Recent studies show that DNA methylation is significantly associated with GM composition and intestinal homeostasis. In addition, GM may also induce histone modification and the immune system (Jones et al., 2015; Ye et al., 2017; Ansari et al., 2020). Microbiota modulation approaches such as fecal microbiota transplantation (FMT) have been helpful in the recovery of abnormal hypomethylation (Ramos-Molina et al., 2019). Probiotic LP’s modulating role in methylation has been studied (Spath et al., 2012; Zhang B. et al., 2023), so probiotic bacteria such as LP may be a useful agent to modulate microbial dysbiosis and associated aging signs.

The evidence of probiotics in aging is growing. Usually, lower animals such as invertebrate *Caenorhabditis elegans* are used as aging models to observe potential bacterial intervention (Derrien et al., 2019; Teame et al., 2020). Probiotic bacteria may influence some drug’s efficacies. For instance, metformin is the most prescribed anti-hyperglycemic drug that acts as a potential pro-longevity molecule due to its key target being adenosine mono-phosphate-activated protein kinase (AMPK). However, feeding metformin to *C. elegans* increases significant lifespan only in the presence of bacteria. Interestingly, Metformin showed no effect on worms when cultured without bacteria or killed bacteria (Cabreiro et al., 2013; Zhang A. et al., 2023).

## 4 *Lactiplantibacillus plantarum* effect on aging hallmarks

Management of aging and associated health burdens has been challenging and complex. In modern medical research, great success has been observed in lowering mortality; consequently, the lifespan of humans has been extended remarkably as the aged population grows rapidly in the modern world (Cong et al., 2021). Aging is a complex, natural process influenced by multiple factors including oxidative stress, inflammation, environmental factors (UV, pollution, and geography), lifestyle, genetics, and epigenetics factors (Rodríguez-Rodero et al., 2011; Trist et al., 2019; Welker et al., 2020).

By managing those factors, the aging process can be improved. For instance, one of the age-promoting factors of DNA methylation, which is susceptible to bioactive nutrients, probiotic supplementation, and gut microbiota composition, may affect the DNA methylation (Halloran and Underwood, 2019; Vähämiko et al., 2019; Allison et al., 2021).

Probiotic bacterial intervention in aging in the early stage is limited to a few preclinical studies. In particular, *Lactobacillus* spp. showed promising effects on the aging model (Ni et al., 2019; Vaiserman et al., 2020). Compounds derived from microbes such as melleolide and microbial invertase may confer rejuvenating and positive effects on several types of aging cells (Gupta et al., 2019; Salekeen et al., 2021). A recent experiment suggests that gut-microbes-associated compounds such as indole-3-propionic acid, dihydropteroate, phenyllactic acid, phenylpyruvic acid, all-trans-retinoic acid, and multiple deoxy-, methyl-, and cyclic nucleotides from gut microbiota are the considerable regulators of NAD+ metabolism and could be the indirect markers for aging-associated degeneration in humans (Rabe et al., 2006; Chen et al., 2023). Fermented soya food items and L-ergothioneine may also provide a series of beneficial antioxidant effects to minimize aging-associated damage (Das et al., 2020). A perturbing consortium of gut microbiota (due to environmental factors, diet, and long-term antibiotics) may weaken the host barrier against pathogens and consequently decrease SCFA production and cause frailty, diabetes, malnutrition, and sarcopenia in the elderly (Nakaya et al., 2014). Probiotics such as LP may help to modulate altered gut microbiota and associated aging hallmarks. Studies suggest that gut microbiota dysbiosis is significantly associated with aging. A recent experimental study in China indicated that a pathogenic alteration in intestinal microbiota composition could decrease telomerase mRNA in mice, while gut microbiota modulation may improve the signs of the aging model (Shenghua et al., 2019). The gut microbiota is one of the most discussed aspects of aging. Altered or disarranged gut microbiota increases age-related disorders, while a healthy composition contributes to longevity and minimization of age-related diseases (Vaiserman et al., 2017; Cӑtoi et al., 2020; Mossad et al., 2022). In addition, the role of organ-specific microbiota could be associated with aging. For instance, skin microbiota composition significantly changes according to age (Li et al., 2020; Vaiserman et al., 2020).

LP’s role in the gut microbiota-associated aging process showed promising antioxidant potential against aging models (Das and Goyal, 2015; Luan et al., 2021). LP can modulate perturbed microbiota and stimulate SCFA levels to build gut microbiota organ-specific crosstalk, which further allows the transmission/activation of activated p38 mitogen-activated protein kinase (MAPKs) and other factors to minimize aging hallmarks such as oxidative stress and inflammation in aging models (Zhang et al., 2019; Lin et al., 2021; Lee et al., 2021a,b).

LP has shown significant antioxidant potential in the D-galactose-induced aging mice model. LP gavage can increase antioxidant activities; reduce malondialdehyde, alanine aminotransferase, aspartate aminotransferase, blood urea, and muscle glycogen levels; and increase overall endurance in mice (Cabreiro et al., 2013; Lee et al., 2021a,b). LP-based probiotic supplementations may influence immune-related genes and toll-like receptor 4 expression. Particularly, *Lactiplantibacillus plantarum* WCFS1 and *Lactobacillus casei* BL23 increased regulatory T-cell frequencies in mesenteric lymph nodes and specific antibody production to support T cell-dependent immune response, which prevents age-related decline in the intestinal mucus of mice (Chen et al., 2017).

**Figure 3:**
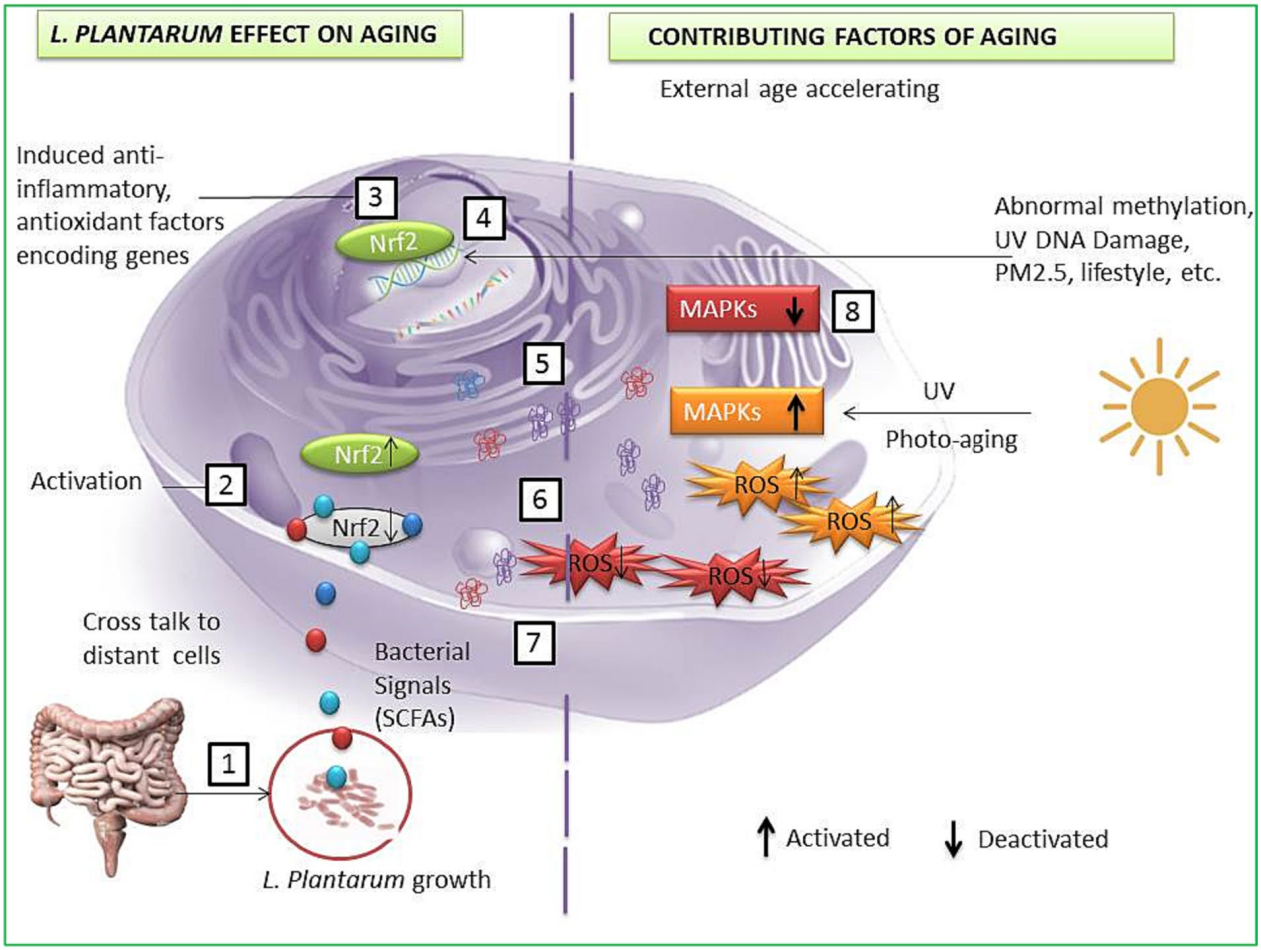
A possible anti-aging pathway induced by *L. plantarum* strains. LP interacts with gut cells and induces signals to other cells such as SCFAs (1); bacterial and host cells crosstalk lead to Nrf2 activation in host cell (2); activated Nrf2 enters the nucleus and activates specific genes to encode enzymes and protein to protect cells from aging factors (3); transcription and DNA repair (4); translated specific anti-inflammatory, antioxidant protein, and oxidative stress-reducing enzymes (5); translocation of cell-protecting proteins and enzymes (6); reduction of ROS (7); deactivation of the MAPKs and other photo-aging and DNA damaging factors (8).

**Figure 4:**
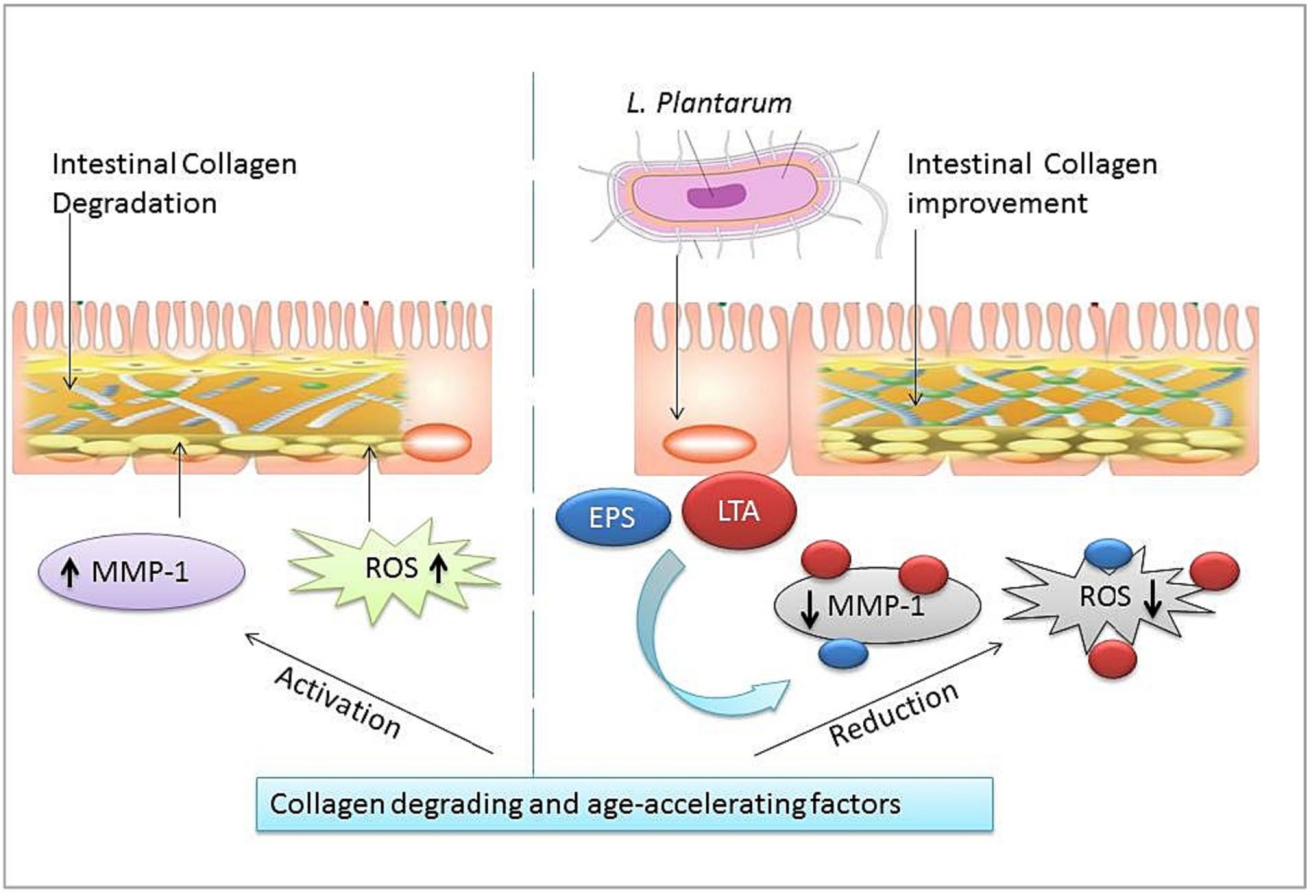
Intestinal collagen and oxidation stress-reducing effect of *L. plantarum* (LP). The right half shows age-promoting factors of matrix metalloproteinases (MMP-1) and reactive oxygen species (ROS). The left half shows LP cell wall constituents: lipoteichoic acid (LTA), exopolysaccharides (EPS) oxidation damage, and collagen degradation reducing effect.

Oxidative stress is one of the most intrinsic factors associated with aging (Tan et al., 2018), and its corollary reactive oxygen species (ROS) can induce the overall aging process including skin aging. Esthetically, the skin is the outermost and largest organ of the human body showing signs of aging directly (Figure 3). Comparatively, the skin aging process may also be accelerated by extrinsic factors such as ultraviolet radiation. Such long-term sun exposure and UV radiation are associated with skin aging known as photo-aging. People with lighter skin are the most sensitive to photo-aging (Sun et al., 2021). UV light exposure may provoke the production of ROS and accelerate photo-aging. ROS hyperactivation causes a signaling pathway that activates matrix metalloproteinases (MMPs) and reduces collagen production. Low collagen level promotes the degrading of connective tissues. On the other hand, induced autophagy and mitochondrial ROS may be able to contribute to aging (Lee et al., 2021a,b; Kumar et al., 2022). Also, a master regulator of redox homeostasis Nrf2 deficiency exacerbates age-related mitochondrial oxidative stress (Chen et al., 2016). A healthy composition of gut microbiota may influence ROS hyperactivation. An oncological study suggests gut microbiota may modulate ROS generation in tumor cells (Kim I. S. et al., 2019). Probiotic intervention has been a promising approach to modulating gut microbiota. Studies show the probiotic antioxidant effect can check ROS and maintain gut homeostasis (Liguori et al., 2018; Yu et al., 2022). In particular, probiotic bacteria LP cell wall constituents such as lipoteichoic and exopolysaccharides can regulate MMP1, MMP2, MMP3, MMP9, and MMP10 expression, decrease the high level of reactive oxygen species, and show promising anti-collagenase and antioxidant activities (Figure 4) (Zhang et al., 2013; Hong et al., 2015; Raza et al., 2017; Shirzad et al., 2018; Kitaoka et al., 2019; Gu et al., 2020; Warraich et al., 2020; Kang et al., 2021; Singh et al., 2022). Therefore, LP supplementation may decrease free radicals and oxidative stress and control the expression levels of TGF-β-related transcription factors in human dermal fibroblasts (Sun et al., 2021). LP also regulates the tight junction in human intestinal epithelial cells and is able to retain moisture in human dermal fibroblast cells by serving as a functional substance in skin-gut axis communication (Lee et al., 2021a,b).

## 5 *Lactiplantibacillus plantarum* associated potential age-ameliorating pathway.

**Figure 5:**
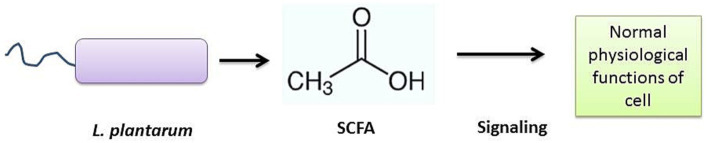
LP-induced SCFA Signaling.

According to the available evidence, LP strains may play an important role in the aging process via gut microbiota modulation, Table 1. LP may help to maintain healthy gut microbiota which maintains intestinal homeostasis by regulating the SCFA and GABA levels (Ni et al., 2019). SCFAs are bacterial signals, crucial to maintain numerous physiological functions. Induced SCFA levels facilitate gut-organ crosstalk, which helps to relocate various essential factors to distant host cells, in order to control cell-damaging factors such as inflammatory responses (Nogal et al., 2021). SCFAs facilitate redox signaling between host and bacterium for normal physiology of the cells (Figure 5).

**Table 1:** Recent findings on the effect of various strains of *L. plantarum* on aging models.

*L. plantarum* strains	Model organism	Key finding	References
*L. plantarum* NJAU-01	Mice	Prevents galactose-induced aging	Ge et al. (2021)
*L. plantarum* KSFY01	Mice	Activation of the Nrf2 improved the athletic ability of mice.	Chen et al. (2022)
*L. plantarum* WCFS1	Mice	Probiotics may influence aging.	Van Beek et al. (2016)
*L. plantarum* GKM3	Mice	Promotes longevity.	Lin et al. (2021)
*L. plantarum* HY7714	Mice	Improves UVB-induced skin damage via the skin-gut axis.	Lee et al. (2021a)
*L. plantarum* JBC5	Roundworm *(Caenorhabditis elegans)*	Promotes healthy aging, gut integrity, and overall lifespan	Kumar et al. (2022)
*L. plantarum69–2*	Mice	Increased SCFA levels alleviate signs of aging via the liver-gut axis.	Wang et al. (2021)
*L. plantarum* ZS62	Mice	Prevents morphological changes in hepatocytes via anti-inflammation and anti-oxidant pathways.	Gan et al. (2021)
*L. plantarum* TWK10	Mice	Improves muscle mass and energy level.	Chen et al. (2016)
*L. plantarum* JBMI F5	Human foreskin fibroblast cell line, Mice	Anti-photo aging (skin aging due to Ultraviolet radiation). Prevents UVB-induced wrinkles.	Kim et al. (2019)

SCFAs may also activate the nuclear factor erythroid 2-related factor 2 (Nrf2) production (González-Bosch et al., 2021). Nrf2 is known to trigger a significant antioxidant pathway in cells. Usually, Nrf2 remains inactive in normal physiologic conditions, while in the activated form, it can enter into the nucleus from the cytoplasm and further activate the antioxidant response element (ARE) to prevent oxidative stress in the host cell. LP supplementation can activate nuclear factor erythroid 2-related factor 2 (Nrf2) in the various types of cells like the skin, liver, and spleen of aging mice (Zhou et al., 2021). Activated Nrf2 may regulate hundreds of genes encoding proteins with anti-inflammatory, antioxidant, drug-metabolizing, and other homeostatic aspects (Figure 2; Liu et al., 2019; Dinkova-Kostova and Copple, 2023). We have discussed that excess accumulation of reactive oxygen species (ROS) is also associated with age-related degenerative disorders such as neurological ailments, chronic sarcopenia, and frailty (Zhang et al., 2017; Hong et al., 2021). LP effect on aging mainly involves the regulation of reactive oxygen species (ROS), stimulation of anti-inflammatory cytokines, and DNA repairing by activating Nrf2 (Tiwari and Wilson, 2019). LP cell wall constituents such as polysaccharides (EPS) are usually responsible for recovering the altered gut microbiota and conferring anti-oxidative effect in aging cells by stimulating enzymes like glutathione peroxidase, superoxide dismutase, and catalase and improving signs of premature aging such as collagen elastin, skin diseases such as atopic dermatitis, and metabolic disorders such as obesity (Zhang et al., 2017; Kim H. R. et al., 2019; Ge et al., 2021; Fu et al., 2022; Yu et al., 2022). Overall, the LP effect against the aging model is positive. LP supplementation can reduce important aging hallmarks such as oxidative stress, inflammation, and mitochondrial dysfunctions via gut microbiota modulation (Figure 2). Although most available research is preclinical, more extensive studies are suggested to explore the possibilities of LP supplementation in aging-associated risk management.

## 6 Conclusion

Human gut microbiota modulation by probiotic LP may influence several aging hallmarks. LP has shown significant antioxidant effects, which prevent oxidative damage in aging cells. Compelling outcomes from available preclinical research suggest that LP strains may induce gut-microbiota-associated signaling such as SCFA and GABA and activate inducible transcription factor Nrf2. Nrf2 induces a cell-intrinsic defense system by regulating multiple specific genes resulting in the production of anti-inflammatory cytokines and protective/antioxidant enzymes (against MAPKs) and reducing ROS. This review suggests LP supplementation may help to manage some aging hallmarks. Yet, significant studies are required to explore LP possibilities to manage the complex aging process.

## Author contributions

NG: Conceptualization, Methodology, Visualization, Writing – original draft. NE-G: Funding acquisition, Writing – review & editing. LM: Validation, Funding acquisition, Writing – review & editing. HG: Investigation, Software, Writing – review & editing. VY: Supervision, Writing – review & editing. SA: Funding acquisition, Writing – review & editing. NQ: Funding acquisition, Resources.
